# To leave or to stay (and what season tickets and club memberships have to do with it)? A qualitative study on the exit of German soccer supporters

**DOI:** 10.3389/fspor.2026.1831306

**Published:** 2026-07-21

**Authors:** Martin Kaden, Axel Faix, Christian Brandt, Gregor Hovemann

**Affiliations:** 1 Department of Sport Economy and Sport Management, Leipzig University, Leipzig, Germany; 2 Department of Management, Dortmund University for Applied Sciences and Arts, Dortmund, Germany; 3 Bayreuth Center of Sport Science, University of Bayreuth, Bayreuth, Germany

**Keywords:** dissatisfaction, fan, loyalty, membership, season ticket, football

## Abstract

**Introduction:**

Fan loyalty is essential to the business model and marketing of professional soccer clubs. At the same time, clubs regularly face fan behavior such as the return of season tickets or cancellation of club memberships. From a sports marketing perspective, these behaviors are often interpreted as indicators of declining loyalty or even fan exit. However, such interpretations tend to oversimplify the underlying dynamics, as the observable behavior does not necessarily correspond to a complete withdrawal from the soccer club. This article examines the extent to which the return of season tickets and the cancellation of memberships at professional soccer clubs can be interpreted as indicators of fan exit, in a partial or total manner.

**Methods:**

An online questionnaire was distributed through German professional soccer clubs and the social media platform *Reddit*. It specifically targeted people who had previously canceled their membership with their favorite club or given up their season ticket. A total of 981 people participated in the questionnaire between October 2025 and March 2026; of these, 198 provided comments on the topic, which were included in a qualitative thematic analysis.

**Results:**

The results are presented separately, depending on whether they include participants’ statements regarding memberships or season tickets. In addition to relevant status constellations, the section also describes various reasons for the cancellation of memberships or season tickets. In the case of canceled memberships, the reasons are largely limited to *issues directly related to the preferred club*. In the case of canceled season tickets, a wider range of reasons is cited, ranging from *an overall negative assessment of the club's offerings* and *a poor quality of the stadium experience* to *logistical problems related to travel to and from the stadiums* and *personal reasons.*

**Discussion:**

The analysis shows that neither the cancellation of memberships, nor the return of season tickets necessarily imply a total exit from the favorite club; however, these actions may be associated with impairments in the fan–club relationship and a partial exit. In this context, implications are discussed within a broader theoretical and management context. Furthermore, the study highlights its limitations and outlines avenues for future research.

## 1 Introduction

For sports marketing in professional soccer, the question of how to interpret changes in fan behavior and loyalty is of paramount importance to the business model. Key performance indicators such as the number of season tickets sold or the development of membership numbers (and the corresponding retention rates) are considered important indicators of fan loyalty and engagement by many clubs. Optimizing ticketing (e.g., concerning the correct ration between season tickets and matchday tickets sold), reducing no-show rates among season ticket holders, and the efforts of many clubs to increase membership numbers (1) have recently received greater attention in both practical club operations and academic discourse. Against this backdrop, this article examines the extent to which two not uncommon phenomena—the return of season tickets and the termination of club memberships—can actually be considered valid indicators of declining loyalty and a fundamental shift away, or exit, from the club on the part of fans or supporters.

This question is particularly relevant for sports marketing because the interpretation of these (and other) behavioral indicators influences decision-making in fan management. If cancellations of season tickets or memberships are interpreted solely as a sign of declining fan loyalty, clubs risk drawing incorrect conclusions about the stability of their fan base. A more nuanced analysis and interpretation of data in fan management, however, reveals that these decisions can be based on various motives or motive groups. These include, for example, negative evaluations of the stadium experience, scheduling conflicts due to match dates, or changes in fans' personal circumstances. Furthermore, normative factors, such as criticism of the commercialization of professional soccer, can play a role in making such decisions—a role that is not always immediately apparent. Considering these potentially diverse motives contributes to a more nuanced understanding of the dynamics of fan behavior in professional soccer and allows for more effective development of fan engagement strategies. If clubs accurately identify the reasons for cancellations, they are able to develop targeted, cause-oriented measures, such as improving the stadium experience or adapting ticket and membership models to the diverse life situations of fans. Insights into normative criticisms can reveal challenges that extend beyond individual clubs and are also relevant for stakeholders at the association level, for example, in the context of match scheduling or the perception of increasing commercialization in professional soccer.

The paper is structured as follows: Section 2 (*Theoretical background*) outlines the underlying model of the study, which is based on Hirschman's Exit-Voice-Loyalty Framework (2), modified by Kaden et al. (3) to apply to soccer and its fan base. The *Literature review* (Section 3) then provides an overview of the current state of research on fan loyalty and exit behavior in professional soccer, including the consideration of season ticket ownership, club memberships, and causes of changes in fan behavior. Following this, Section 4 (*Materials and methods*) discusses the data collection and analysis procedure based on a qualitative approach. The empirical findings are then presented in Section 5 (*Results*) and systematically related to the underlying research question. In the subsequent *Discussion* (Section 6), the results are placed in a broader context while answering the research question. Concluding, management-related implications for the marketing of professional soccer clubs, limitations of the methodological approach, as well as theoretical implications and starting points for further research on fan behavior in professional sports are derived.

## 2 Theoretical background

As the theoretical background, this study is based on the Exit-Voice-Loyalty framework of Hirschman (2) in its basic form, as well as of Kaden et al. (3) in its modified form, adapted to the research subject of soccer and its supporter base.

In its basic form, the framework describes two response options available to members of an organization or customers of a company in the event of dissatisfaction, whether with the products or services offered or with the leadership or management of an organization: *Exit* and *Voice. Exit* describes the end of any form of involvement (4), for example, when members of an organization terminate their membership or when a company's customers stop purchasing its products or using its services. Briefly, Kiernan (5) marks the exit as a “cut and run” (p. 884) response. *Voice* is the alternative option for members/customers who do not turn away but, as Kiernan (5) puts it, “stay and fight” (p. 884). For example, one can speak of the use of the voice option when dissatisfied customers contact a company via complaint hotlines or when protests against the management of an organization are raised at general meetings. In this respect, the voice option expresses an attempt to bring about change, if not to force it, as Nikolychuk and Sturgess (6) explain. Completing the framework, the factor of *Loyalty* plays an intervening/moderating role between the possibilities of exit and voice (4, 5) by appearing, as Hirschman (2) puts it, as a “special attachment” (p. 77) of customers/members to a company/organization. This feeling of particularly strong affection or sympathy prevents dissatisfied customers/members from turning away. Instead, they stay and make greater use of the voice option and, again in the words of Kiernan (5), *fight* for something to which they are so particularly attached (4, 6, 7). In this respect, loyalty prevents an exit by acting as a “barrier” [(6), p. 843] or, at the very least, causes a delay as a “brake on the decision to exit” [(2), p. 88].

Hirschman's (2) framework has already been applied repeatedly concerning soccer and its supporter base (e.g. 6–10), with Kiernan (5) describing it in this context as “particularly useful […] to apply to the ebb and flow of modern football” (p. 880). Kaden et al. (3) justify the advantageous and appropriate use of the framework, as it allows for an explicit focus on supporters and their perspectives in an environment that is becoming increasingly disadvantageous for them. For example, when tendencies toward increasing commercialization are accompanied by higher costs for supporters, or when their voices, opinions, or expectations are fundamentally given less weight in favor of economic interests. The work of Kaden et al. (3) is also relevant to the present study in that it adapts Hirschman's (2) understanding of the exit reaction to the field of soccer and its supporters. Thereby, an exit is differentiated between a *total* and a *partial* form and defined as follows, with this modified understanding also generally used as a basic point of reference in this study:

A “total” exit in the context of soccer supporters refers to the complete cessation of support for a previously favoured club. This exit is final in terms of time, that is there are no plans to resume the former support. Original behavioural loyalties and emotional-psychological connections/bonds to the former favourite club are entirely dissolved. A “partial” exit is also characterised by this finality in terms of time, but in this case, the supporters do not completely stop supporting their favourite club. Impairments/cracks/erosions in loyalty/bonds are permanent and reflected in the (emotional/psychological) attitude and behaviour of supporters towards the club, but the relationship is maintained, at least in part [(3), p. 15].

**Figure 1:**
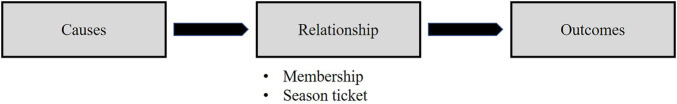
Fan–club relationship.

Partial and total exits share the fact that they affect the *relationship* between fans/supporters and their clubs. Basically, the relationship between fans and a club is characterized by specific behaviors and attachments, based on particular causes, and leads to specific outcomes (for fans and clubs as well). However, they differ in their radical nature, and the causes of a partial and a total exit can overlap. The further discussion refers to the following model (Figure 1), considering the perspective of fans. It characterizes the relationship between fans and clubs by fans' membership in a club and their regular visits to the stadium due to a season ticket.

It should be noted that Kaden et al. (3) use the term *supporter* in a rather neutral way, without attributing a specific level or degree of loyalty to this term from the outset or suggesting that such a level or degree exists. The reason given for this is that terms used to describe people who, fundamentally speaking, have a connection to a soccer club (in whatever form), such as supporters, followers, fans, spectators, customers, or consumers, “are often used undifferentiated, interchangeably, and thus inconsistently” (p. 4). This is despite the fact that typologies and taxonomies, the best known of which is probably that of Giulianotti (11) with its differentiation between flâneurs, fans, followers, and supporters, point to corresponding differences in terms of these groups' level of loyalty to a particular soccer club [an overview of other traditional segmentation approaches is provided, for example, by Bodet (12) or García & Llopis-Goig (13)]. The quote from Rookwood and Chan (14), who speak of a “blurring of distinctions between supporter types” (p. 902), is also aptly cited in this regard. This study follows this basic understanding of Kaden et al. (3) and the explanations provided therein, whereby the terms *supporters* and *fans* are used impartially and fundamentally encompass all followers, fans, spectators, customers, consumers, etc. of soccer clubs, without any a priori assumptions regarding stronger or weaker degrees of attachment to respective soccer clubs in this context.

## 3 Literature review

Kaden et al. (3) provide a basic overview of the field of research in their scoping review on the exit of soccer supporters, although only a marginal number of relevant studies on the topic could be identified. In this context, the authors observe that this number does “not initially suggest that this is an intensively researched topic to which researchers have given priority or are devoting increased attention” (p. 14). Furthermore, it seems obvious in this regard that this very limited discussion of the topic goes hand in hand with what Tapp (15) describes as the conventional, traditional, or simplified assumption that soccer fans support their favorite clubs “unstintingly loyal [or] evermore” (p. 203) and that further discussion is therefore unnecessary or takes place in a rather marginal way. This usually considered high level of loyalty continues to reinforce the assumption that soccer club supporters prefer the voice option in cases of dissatisfaction (see Hirschman (2) himself, and also (16–18)) and that an exit reaction, if it occurs at all or can be observed, simply offers less cause for research.

In this context, various studies can be cited which, although they point to cracks in the relationship between supporters and their favorite clubs, cannot confirm or question a final break in the bond [following Kaden et al. (3), i.e., studies that point to a partial but not a total exit]. For example, Watkins and Cox (19) argue that fans are quite capable of turning away from their favorite professional clubs, at least to a certain extent, and supporting an amateur club, with “disillusion with modern football” [in this connection, the issues of “commercialization, erosion of competition, stadium experience, devalued as fans” (p. 5) are cited as factors] as decisive in this regard. However, a complete exit is considered questionable: “The fans we interviewed have not and may not ever relinquish support for their professional team” (p. 12). Busse and Damiano (20) argue that progressive commercialization is gradually having a negative impact on the emotional relationship and loyalty between fans and clubs, but that support is nevertheless being maintained. However, the relationship with soccer in general is already being damaged: “Among the interviewed fans, some stated that the interest had already decreased and that the focus is more on the club itself” (p. 27). Both Healy and McDonagh (21) and Tinson et al. (22) refer to an exit from the market, whereby supporters, for example, boycott stadium visits or feel excluded from them, but remain part of the supporter community (“Exit from the community is not a much used option given the strength of fan loyalty to their community identity” [(21), p. 1535]), or find ways to compensate (“There were no fans in our sample who had entirely exited the market. Although fans reduced their nature and level of participation in the market, they were reluctant to disassociate completely from their team. This supports the notion that fans are able to compensate perceptions of value being disrupted by trading-off negative experiences with ones that allow value to be recovered” [(22), p. 11]).

In addition to shortcomings concerning the attractiveness of soccer (e.g., in terms of performance on the field or the atmosphere in the stadium) and the governance and management of soccer clubs, personal factors related to the individual circumstances and situations of supporters are cited as causes in this context (3, 21, 22). Conceptual approaches in this context are provided by Tamir (23) and Bodon (24). Tamir (23) describes a dynamic *life cycle for sports fans*, in which the relationship with the favorite club is more or less pronounced at different stages, but nevertheless continues to exist: “Theoretically and practically, fanhood neither begins nor does it end at a specific point in time” (p. 1). In his extension of the Psychological Continuum Model, Bodon (24) concludes that a process of disattachment from soccer teams ultimately results in distance, but not in complete non-existence or a complete loss of attachment, “because the peculiarity of the fan's emotional world is that attachment does not disappear without a trace” (p. 17).

There are only a very limited number of results available on soccer fans completely turning away, although Hie et al. (25) point out in their study that both the unusual behavior of fans switching to another club and a total exit is possible: “Some fans begin engaging with other football teams, while some can move away from football entirely and focus on other aspects of their lives, unsure if they would return” (p. 1). Similarly, Kaden et al. (7) refer in their study to some former supporters who, by their own admission, have permanently turned away from their previously favored clubs, “with some reporting they found substitutions for their support in and outside of the sport of soccer” (p. 12). Putra (26) reports on fans “systematically excluding themselves from the fandom” (p. 64), citing the example of a former Liverpool FC supporter who not only stopped supporting the club, but now also opposes his former favorite team. In the context of these studies, issues are also raised that concern, among other things, the commercialization and the attractiveness of soccer, as well as the governance of clubs (7, 25, 26). Furthermore, Hie et al. (25) conclude that supporters can become divided over issues or how to address them in disagreements, lose contact with one another, and end up feeling aversion, mistrust, or superiority toward each other, or even resort to violence against each other. According to Kaden et al. (3), a “mutual influence between fans” (p. 13) appears to be a factor that potentially provides further reasons for turning away or reinforces this response.

Concerning supporters of a soccer club as season ticket holders or members, it should be noted that existing studies have not yet addressed the issue of exit [as defined by Kaden et al. (3)], or have only touched on it in a broader sense, and/or tend to reinforce the traditional image of the loyal soccer supporter. Faix (1) conceptually considers the core tasks of member management of professional soccer clubs and distinguishes between acquiring new members, maintaining and developing existing member relationships, and preventing member termination (and, if it seems appropriate, winning back lost members). Concerning the last task bundle, the establishment of a monitoring system is proposed, which records and processes indications of growing discontent among fans with decisions of club management and other factors of interest, and helps to counteract possible exits of members. Bauers et al. (18) describe German soccer club members as emotionally attached, loyal, and particularly interested in their respective clubs, precisely because their membership gives them influence and decision-making rights (if intended to help protect the values and tradition of a club in the face of massive commercialization), guaranteed and secured by the so-called *50 + 1* *rule*^1^. Furthermore, instrumental motives may be relevant for fans, such as access to pre-sale rights for tickets or improved chances of obtaining season tickets. Similarly, studies on season ticket holders suggest that these are the most loyal and fanatical supporters of clubs, with a strong sense of identification and close emotional and behavioral connection, a feeling of belonging (to a club as well as to a community of fans), and a way of life that is structured around and aligned with their favorite team; with reference to a potentially diverse range of motives for purchasing a season ticket, price advantages and the safety, to be able to see every home game, can also be relevant (27–32). Several studies have examined the (dis)satisfaction of season ticket holders in sports and the factors that influence it, indicating that the *price-performance ratio* plays a decisive role in the valuation of such services [i.e., (33, 34)]. The relevant factors can be incorporated into the monitoring system mentioned previously for the early detection of the need for action in the membership context.

Against this backdrop, this study is based on the premise that a terminated membership or a canceled season ticket may represent an act of reduced loyalty or even an exit, but requires intensive research and in-depth analysis to obtain a valid assessment in this regard. Therefore, the research question for this study is as follows: *Can canceled season tickets and memberships be linked as indicators with the exit of German soccer fans? And what reasons determine whether the question is answered in the affirmative or in the negative?*

## 4 Materials and methods

For the purpose of data collection, a questionnaire was created and distributed in the context of German soccer and its supporters, which, in addition to sociodemographic data (age, gender), also recorded whether the participants have or had a favorite soccer club and, if so, which one. Furthermore, questions were asked about membership status in the favorite club and season ticket ownership. Participants could choose whether they are currently club members/season ticket holders, had been club members/season ticket holders in the past, or neither, i.e., had never been club members/season ticket holders and were not currently either. As part of an open-ended question, participants were also allowed to make further comments on the topic and, if applicable, on their specified (potentially former) favorite soccer club. The answers to this open-ended question form the data corpus of the qualitative analysis.

The questionnaire was distributed in two ways. Firstly, professional soccer clubs in the Bundesliga (Germany's top soccer league), the 2nd Bundesliga, and the 3rd League were contacted and asked to share the questionnaire with people who had either terminated their club membership or canceled their season ticket. Secondly, the questionnaire was shared on the social media platform *Reddit*. The platform itself is characterized by its high number of active users and therefore offers the advantage of a wide reach and the acquisition of detailed content with great depth and substance. In addition, the platform offers the opportunity to distribute a request in various so-called subreddits, i.e., specialized communities with specific areas of interest (35, 36). Another advantage is the anonymity of users emphasized by Reddit, which, as Jha et al. (35) explain, is “fostering open expression of opinions without apprehension” (p. 6). The questionnaire was shared in separate club-specific subreddits, where supporters of respective clubs in the Bundesliga and 2nd Bundesliga, as well as the 3rd League, are active as users. As part of the request to participate, users were asked whether they had (ever) canceled their season ticket or terminated their membership in their favorite club.

The data collection was conducted between October 2025 and March 2026, i.e., during the 25/26 soccer season. In accordance with the ethical guidelines for internet-based research, and following Convery and Cox (37), the survey was conducted completely anonymously. Insofar as real names or pseudonyms (or even the names of clubs or cities) were used in the context of the open-ended questions, these were removed during the analysis. Users who wanted to participate in the survey had to agree to a mandatory privacy policy beforehand.

The survey included 620 male, 50 female, and four diverse participants; 307 participants did not specify their gender. Concerning the age, the following numbers of participants were recorded by age group: 18–25 years: 88 participants; 26–35 years: 284 participants; 36–45 years: 148 participants; 46–55 years: 82 participants; 56–65 years: 40 participants; 66 years and older: 12 participants (No information provided: 327 participants).

A total of 198 responses to the open-ended question were included in the qualitative analysis. The responses were first reviewed jointly by the first two authors in order to agree on a basic approach for further independent qualitative analysis of the material. It was fundamentally agreed that only those responses that allowed comprehensive conclusions to be drawn, with regard to answering the research question, would be included in the analysis as relevant. This means that responses were included as relevant if both a *status* regarding the season ticket and/or membership was specified (e.g., season ticket not renewed/membership terminated) and further *contextualization* took place (e.g., by stating reasons why the season ticket was not renewed/membership terminated). The subsequent coding and analysis aimed at a holistic view of these levels accordingly. The approach was based on the *Thematic Analysis* according to Braun and Clarke (38), with the proviso that from the available material, initial themes and codes were generated, “which *might* provide a meaningful *answer* to the research question” (p. 35). Consequently, all 198 responses were first reviewed in parallel by the first two authors using *MAXQDA*, a software for computer-assisted qualitative analysis, independently coded, and searched for potential themes.

Subsequently, to increase the reliability of the procedure, all initially generated results were reviewed and evaluated together, and recoding was carried out where necessary. In this regard, the approach according to Braun and Clarke (38) should be understood as a non-linear but recursive and iterative process, which is also characterized by an “ongoing reflexive dialog on the part of the researcher or researchers” [(39), p. 82]. The non-sole or non-exclusive analysis of a single researcher within the scope of this study can, following Braun and Clarke (38), also be described as “collaborative coding […] used to enhance understanding, interpretation, and reflexivity, rather than to reach consensus about data coding” (p. 8). Finally, the resulting topics and assigned codes were named for clarification purposes and provided with definitions. Concerning the information gathered by the participants about possible causes for the cancellation of season tickets and club memberships, the aim was, as far as possible, to identify corresponding systems of reasons by means of inductive reasoning, in which general patterns are recognized bottom-up from the observation of individual cases (which subsequently could play a role when it comes to further theory development).

In order to provide a well-founded and rigorous answer to the underlying research question, the final coding scheme was designed to systematically capture membership and season ticket status, as well as additional relevant context. Status information was coded separately depending on whether it referred to club membership or season ticket ownership. Similarly, the overall tone was coded separately, depending on whether the relationship with the favorite club tended to have negative (e.g., threatening to cancel membership due to dissatisfaction or returning a season ticket out of displeasure) or positive (e.g., gaining membership or a season ticket; expressing support for the club) connotations. To provide further context, the reasons for a negative or positive attitude toward the favorite club were also coded separately in this regard. Comments that were merely one-sided remarks regarding status or context were coded but not included in the subsequent analyses, as they did not allow for a comprehensive conclusion.

## 5 Results

Below, the study's results are presented. With reference to the underlying research question, the results regarding the status and context of season tickets and memberships are presented separately. Thereby, a brief numerical overview of the data on season tickets and memberships is provided. In the following analysis, concise, meaningful statements from the participants are cited as examples (the location in the data material is indicated by the abbreviation *P (Participant)* followed by a number). Care was taken to translate the participants' comments in a way that preserved the original content and tone.

### 5.1 Membership

The study reveals the following statuses regarding club membership: I am a member of the club: 506 participants; I was a member of the club: 68 participants; I have never been a member of the club and am not currently a member: 316 participants (No information provided: 91 participants).

First, regarding membership, it should be noted that none of the participants in the sample directly mention giving up their membership as part of a complete break with, or total exit, from their favorite club. For example, one participant explicitly mentions dissatisfaction with the club and the scheduled termination of his membership, while at the same time affirming his identity as a fan: “That's why my membership will end on June 30, but I'll still be a fan” (P281). Furthermore, some participants state that they are considering or have considered canceling their membership, but have not (yet) done so. In such cases, the decision to cancel is subject to certain conditions, such as a specific sporting performance—“If the [club] is relegated, I will consider renewing my membership” (P351)—or certain events. One participant reports that membership was only retained because of a perceived dissonance between one's own identity as a fan and identification with the favorite club was resolved: “It was only after a credible change in this matter that the withdrawal from membership was not carried out” (P1068). A participant (P409) expresses a similar view, and although he takes a critical stance toward “his own club, which is increasingly bowing to the conventions of professional soccer, thereby becoming arbitrary and losing its identity”, and also voices “criticism of the structure of professional soccer in general”, he nevertheless maintains his membership (for the time being): “It is only my deep attachment to the “old” club (the first team is now a limited liability company) that is stopping me from canceling my membership. But really, it's only a matter of time.” Another participant, who had to return his season ticket for health reasons, sums up the unbreakable bond with the club that his membership symbolizes as follows: “The only thing that will end my membership is my death” (P281).

The reasons for (potentially) giving up membership, as evident from the statements cited, all share the common feature that they are directly related to the club, its community, and identity, and that the (potential) ex-members express a feeling that they no longer or should not belong to that, while having, as members, a certain responsibility for the development of the favorite club. Thereby, the reasons are linked to a certain degree of *dissatisfaction with the club's management or developments at club level*, and can, in short, be described as *club-related reasons*. One participant, for instance, criticizes club policy in general for creating a “growing distance from the fans” (P281), whilst another participant echoes this view of increasing distance, stating that “people are drifting further and further away from their clubs and, as in my case, turning back toward amateur soccer” (P404). P351 criticizes the club's management directly in relation to the team's sporting performance, the transfer policy, and its scouting and youth development work, stating: “I am dissatisfied with the work of the sporting management!” Another participant's statement is notable for the fact that it directly criticizes not only the club's management but also other groups of fans and the way they are treated: “The relationship with the club has been massively influenced by former officials and fan groups”. In particular, the criticism here is that the club merely responds to “groups and individuals who are prone to violence and hold extreme right-wing political views” with a “lack of credible rejection” (P1068). In conclusion, the identified club-related reasons for dissatisfaction can be classified, on the one hand, into measures and decisions of the club management that *affect the club's core activities* (transfer or financing policy, ticketing, dealing with fan affairs, etc.). On the other hand, the focus is on the *club's fundamental social positioning*, expressed, for example, by the dealings of those in charge with the misconduct and misguided views of one's own supporters.

### 5.2 Season ticket

The study reveals the following statuses regarding season tickets: I have a season ticket for the club's matches: 196 participants; I used to have a season ticket for the club's matches: 248 participants; I have never had a season ticket for the club's matches and do not currently have one either: 447 participants (No information provided: 90 participants).

When discussing season ticket arrangements, participants generally make a wide range of statements regarding their current status. First, there are statements that directly indicate that participants no longer hold a season ticket, such as “I have been living in Colombia since October 1 and therefore no longer have a season ticket” (P383). Comments from other participants suggest that a season ticket may no longer be used, or may only be used for a limited number of games; for example, P561 mentions: “Every now and then, I wonder if I could make better use of my time than spending it at the stadium”, or P677 (who also mentions options for compensation): “I've often thought about only going to the second team's games, or to the nearest club that plays in the district league”. The issue of reduced usage is directly addressed by P728: “I'm using my season ticket less and less”, and P799: “I have three season tickets in the standing section. But these days I only go to at most seven league games (against decent opponents who bring good fans)”. Similar to the remarks regarding membership, however, it is also worth noting statements made by participants in the context of season tickets who, while confirming that they no longer hold such a ticket, at the same time emphatically affirm their status as fans. As noted by P374, the club is explicitly excluded from the decision-making process (“The fact that I no longer bought a season ticket has less to do with the club”) or personal reasons are cited, as in P310: “I only gave up my season ticket because I moved 600 km away from [city] for personal reasons. I'll always remain a fan of the [club]”, or P360: “I only canceled my season ticket because I can no longer go to the stadium for health reasons. That's the only reason. In my heart, I am and always will be a fan”. Partially, in this context, conditions are also set forth which, once met, guarantee the reusage of the season ticket, e.g., due to temporary health issues: “Unfortunately, I had to give up my season ticket because my health isn't up to it anymore. I'm currently under a doctor's order not to drive; once that's lifted, I'll be back in the stadium” (P409), or the hiring of an unpopular coach, with P490 stating: “As soon as he's gone, I'll be back!” Furthermore, the sample includes participants who would like to obtain a season ticket but have no prospect of doing so; one participant speaks with resignation: “I think the [club] stopped accepting new names on the waiting list for season tickets a few years ago because demand is so high and they want to sell a certain percentage of seats (including standing places) through single-game tickets” (P764).

The comments on the questionnaire provide a detailed account of the various reasons for giving up season tickets. Following a thematic analysis of the comments, they were classified into the following inductively derived categories: *overall negative assessment of the club's offerings, poor quality of the stadium experience, logistical problems related to travel to and from the stadiums*, and *personal reasons.* Compared to the reasons for (potentially) giving up memberships, it is apparent that the arguments surrounding season tickets are influenced more by personal considerations and dissatisfaction with certain aspects of one's (individual) soccer experience, rather than solely by the management of the respective favorite club.

The *overall negative assessment of the club's offerings* stems from various factors, among which the lack of suitable ticket options (e.g., no tickets valid for only six months) and high (increased) costs associated with attending games (ticket prices, food and beverages, etc.) directly impact the core aspects of the offering that are crucial to the price-performance ratio from the fans’ perspective. For example, P1043 states: “Above all, the prices of tickets, food, and drink at the stadium make attending a game a luxury that many people can no longer afford”. Referring directly to season ticket prices, one participant commented: “Unfortunately, the season tickets were far too expensive to sit in Section 5. I just can't afford it” (P431). Furthermore, assessments of the price-performance ratio seem to reflect broader, critical views of trends in professional soccer toward increasing commercialization, with P677 noting in short: “It's all just commerce and business as far as the eye can see”. Another participant, who used to hold a season ticket, cites not only “increasing commercialization” but also dissatisfaction with the direction taken by the club's management as reasons for criticism, noting that “supporting the team actually started to feel like being just a *customer*” (P517). Furthermore, in this context, there are complaints about spin-off processes, with one participant remarking that “since the spin-off […] I've been nothing but a fan of the fan block [the Ultras’ standing areas]” (P799). Referring primarily to economic interests, another participant (who has decided not to renew his season ticket for a second-division soccer club but retains his season ticket for a local handball club) comments on the players' attitude: “At least the guys [the handball club's players] always give their best and never complain! That has to do with honest sportsmanship and dedication! I miss that in the current players [of the soccer club], who, of course, are mostly just mercenaries passing through and hardly identify with the [club]” (P351).

The *poor quality of the stadium experience* encompasses factors that can negatively impact the stadium visit in various ways. Participants cite risks posed by pyrotechnics, aggressive fan behavior, or alcohol-related conflicts; one participant notes: “In fact, I've been distancing myself more and more because the increasing violence of the Ultras really put me off […]. Soccer is a great sport, but the atmosphere in the stadium is ruined by drunken idiots who are losing their inhibitions more and more and grossly overestimate their infuence on the game or the atmosphere” (P591). In this context, the behavior of other fans—both at home and away games—is perceived as “embarrassing” (P338, P591), and the sense of entitlement or smugness of such fan groups is criticized, who “think the stadium belongs to them and they can do whatever they want” (P357). P728 makes a further distinction among fan groups and, under the broad umbrella of “disturbing trends in fan culture”, laments on the one hand “overly organized Ultras”, and on the other hand “newly arrived *event* fans” who negatively impact the mood and atmosphere in the stadium: “I increasingly miss the individual, grown enthusiasm that, for me, used to define the stadium experience”. Additionally, participants raised complaints about the negative impact on the stadium experience caused by smokers and obstructed views due to fans standing in the aisles.

*Logistical problems related to travel to and from the stadiums* provide further reasons for participants to return their season tickets. The analysis of the comments reveals that, in particular, inconvenient and/or unreliable public transportation and train connections, as well as inadequate parking facilities, significantly hinder fans' ability to arrive at and depart from the stadiums on time. For example, P427 points to the responsible regional transport company and states: “Buses to the game always run too far apart. Every 20 minutes is too long. The stops in the city are always crowded”. Such problems often have a cumulative effect, particularly with evening and weekday games that conflict with work and family obligations. In this regard, the scheduling of games is also criticized, e.g., by P313: “I decided against getting a season ticket because, unfortunately, I can't attend every game. Sunday game schedules aren't ideal, especially for commuters” or P439: “The impossible kickoff times on three different days make it nearly impossible for me to watch every home game. I live in [city]. The trip to the venue is 300 km, and if you work on Friday and Sunday, games are already out of the question 90 percent of the time. And if you can only use a season ticket 50 percent of the time, you start to think twice and have to make a decision”.

In connection with these logistical barriers, however, it should be noted that they are often linked to *personal reasons* or life changes that no longer allow people to attend games (as they used to). A change of residence is frequently cited as a reason (see P310 and P383 above), which increases geographical distances, impairs previously established mobility routines, and thus calls into question the benefits of season tickets; see also P347 as an example: “The main reason for not renewing the two season tickets is the now even greater distance to [city]. In addition to the previously existing travel of over two hours from home, two out of five family members have now started studying, adding significantly more travel time, which makes more frequent attendance at games impossible”. In addition to work commitments, family obligations are cited as factors that limit flexibility and make attending games more of a hassle: “Between my child and my job, going to the stadium is extremely difficult. Not to mention away games” (P873). Health issues or illness are also frequently cited as additional reasons for returning season tickets, which fall within the personal sphere of the participants (P276; P392; P32). One participant explains that such reasons do not necessarily mean turning away from the club or soccer: “The [club] is like a second family to me. I've always felt very comfortable at the stadium. Unfortunately, due to health reasons, I can no longer attend the stadium. I watch the games from home. And through some fan clubs, I'm always well-informed” (P314). Furthermore, P378 observes social changes in his own life—in this case, within his group of friends and acquaintances—that could potentially cause fan communities to fall apart and make the use of season tickets seem problematic: “I didn't renew the four season tickets I had before because there was increasingly less interest among my friends and acquaintances in going there with me. That's why it became a struggle for me every matchday to find people to take the tickets. […] I live within walking distance of the [club's] stadium and used to enjoy going there on weekends to watch soccer with friends, but that was becoming more and more of a problem”.

## 6 Discussion

The following discussion starts by addressing the research question formulated, from which theoretical and then practical (management-related) implications are derived. Furthermore, the study's limitations are discussed, and suggestions for future research are provided.

### 6.1 Answering the research question

Can canceled season tickets and memberships be linked as indicators with the exit of German soccer fans? And what reasons determine whether the question is answered in the affirmative or in the negative? The answer to the research question builds on the distinction made in the definition of exit by Kaden et al. (3) between a total and a partial exit; however, it is important to note that neither the cancellation of season tickets, nor the termination of memberships, inevitably constitutes an indicator of an exit. These two types of behavior can occur in various contexts and for a variety of reasons; however, they do not necessarily have to be associated with the two types of exit, but can, in principle, also occur within intact relationships between clubs and fans.

Concerning the termination of memberships, only a relatively small proportion of the participants (68 participants) reported having been members of their respective favorite club. None of these persons, however, make any remarks linking the termination of their membership with a total exit; instead, they affirm their status as fans and their continued connection to their favorite club. This finding basically reinforces the traditional image, as discussed by Tapp [(15); see also the conceptual approaches of Tamir (23) and Bodon (24)], of the unconditionally loyal soccer fan who maintains an unbreakable bond with their favorite club; in this context, the statement by P281 can once again be cited as an example, clarifying that his membership is terminated solely by his death. It should be noted, however, that those participants in the sample who stated that they wanted to terminate their membership (at least once) can certainly be considered to have made a partial exit, as such statements suggest ruptures or cracks in the fan–club relationship (3). As noted by Busse and Damiano (20), (behavioral) support for the favorite club then remains intact, even though the relationship is subject to negative influences and deterioration on an emotional level. In short, terminating one's membership does not necessarily mean—or automatically imply—that one is turning away from one's favorite club. Nevertheless, attention should be paid to membership status and any actual or emerging changes in that status, since, as Bauers et al. (18) note, membership—as an expression of emotional attachment and loyalty—allows one to draw conclusions about a potentially deteriorating state of the relationship between fans and their favorite clubs. In this regard, a (potential) exit of a fan might be understood as a longer process, where the termination of the membership is only one potential step.

As for the reasons for (potentially) canceling memberships, it is notable that participants provide a much more limited range of justifications, reasons, and related circumstances (compared to the corresponding statements regarding season tickets). Nevertheless, both in the context of memberships and season tickets, it can be assumed that the original motives for holding them might lose some or all of their validity to the extent that corresponding reasons become decisive (see also the explanations in the literature review of this study in this regard). Essentially, in the context of memberships, the focus is (solely) on negative changes in the relationship between the club and its supporters, as well as dissatisfaction with the club's management. This is understandable insofar as members of German soccer clubs generally have a direct degree of influence and a right to participate in decisions regarding the affairs of their respective favorite club and its development (18). Given this situation, which also involves the payment of membership dues to the club, it is reasonable to assume that paying members have certain expectations or entitlements regarding the club's management, and that any deviation from these expectations can lead to a certain degree of dissatisfaction [see also Hirschman (2) for a fundamental discussion of this topic]. This finding is also consistent with a rather notable number of studies on soccer and its supporters that address issues related to the governance (such as perceived mismanagement) of favorite clubs [e.g., (3, 7, 21, 22, 25)].

**Figure 2:**
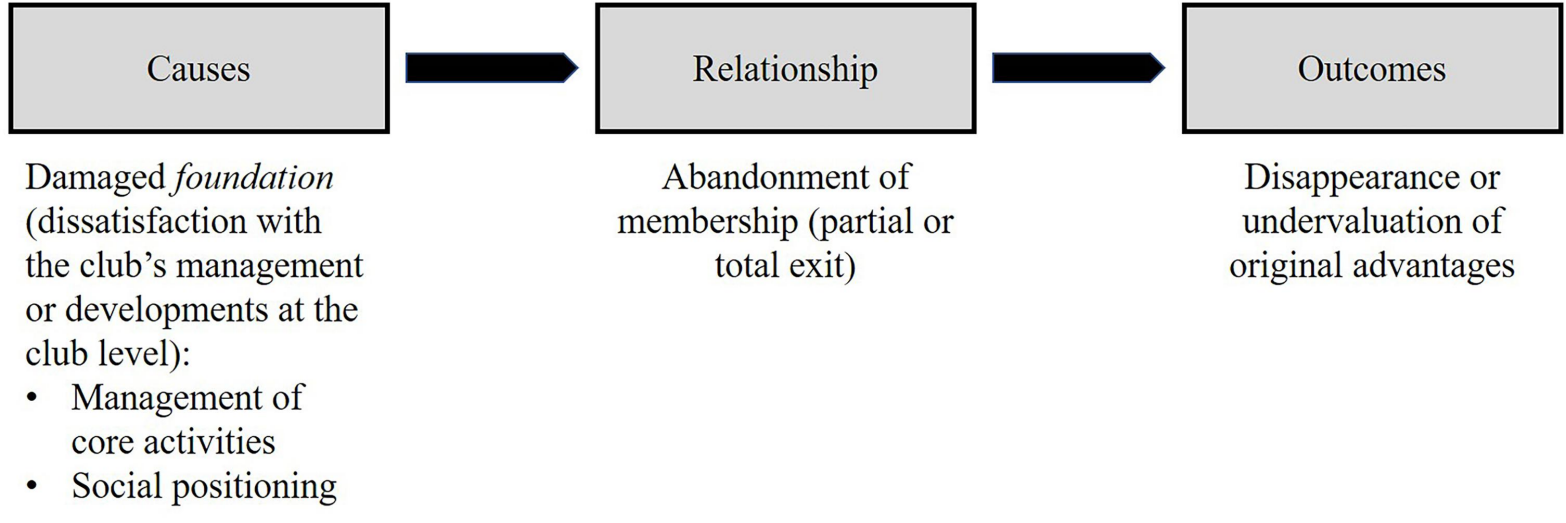
Fan–club relationship and abandonment of membership.

The following overview summarizes the findings regarding membership against the background of the relationship between fans and clubs (Figure 2). Although this should not necessarily be interpreted as a total exit, the findings show that abandoning a membership is, in principle, caused by the devaluation or disappearance of former causes and/or the emergence of disturbances. These reasons are likely to be damaging to the foundation, or, as a rule and in the truest sense of the word, of a *fundamental* nature.

When analyzing the statements regarding season tickets, it is first worth noting that, compared to the recorded number of canceled memberships, a larger proportion of the 248 respondents indicated that they had canceled such a ticket. Nevertheless (as was also the case with the identified instances of membership termination), none of the participants' statements in this context suggest a total exit. On the contrary, participants repeatedly emphasize their loyalty and the fact that their relationship with their favorite club remains intact, noting that giving up their season ticket and the reasons for doing so have nothing to do with their identity as a fan, or that they will definitely renew/reuse their season ticket as soon as the necessary conditions are met. Considering this, the cancellation of season tickets may be triggered by changes/reasons, although the relationship with the club remains fully intact. In addition, the sample includes participants who have apparently not held a season ticket to date but state that they explicitly intend to purchase one. Once again, the image discussed by Tapp (15)—of the ever-loyal soccer fan who supports their favorite team no matter what—comes to mind. These findings are consistent with the results reported by Healy and McDonagh (21) and Tinson et al. (22), according to whom fans may withdraw from the market or feel excluded from participating in it, yet continue to feel a sense of belonging to their favorite club and the community of supporters. However, concerning season ticket status too, it should be noted that some participants in the sample made statements suggesting a partial exit from their respective favorite club (3), which is linked to a lack of opportunities and/or motivation to attend games (regularly), which in turn results in reduced support for the club. Thus, an impaired fan–club relationship is indicated at the behavioral level when participants report that they are using their season tickets less and less frequently (see the corresponding statements by P728 and P799). The same potentially applies on an emotional level when fans intend to use their season tickets less frequently or cancel them. At this point, it is worth referring once again to the statements of two participants who underscore this, as they are also actively considering alternatives to supporting their favorite club [see the corresponding quote from Kaden et al. (7) in chapter 3 as well] P561 wonders whether he “could make better use of [his] time than spending it at the stadium”, and P677 has already “thought about only going to the second team's games, or to the nearest club that plays in the district league”. The last statement also draws direct parallels to the work of Watkins and Cox (19), who cite support for lower-division or amateur clubs as a potential compensation for disillusioned soccer fans. In summary, it should also be noted regarding the status of (canceled) season tickets that, while this does not necessarily imply an exit from one's favorite club, a closer analysis may sometimes reveal signs of a relationship between fans and their favorite clubs that has been impaired to some extent.

Compared to the participants' statements regarding membership in their favorite clubs, a much wider range of reasons, justifications, and circumstances is cited when it comes to canceling season tickets. Concerning the overall negative assessment of the club's offerings, fundamental connections are repeatedly drawn to the increasing commercialization of soccer, a key issue criticized by fans that is also addressed in a number of other studies [e.g., (7, 19, 20, 26)]. A telling example here is the connection between the statement cited from P517—who increasingly sees his role shifting from that of a supporter to that of a mere customer of his club—and the study by Watkins and Cox (19), which highlights a devaluation of fans and growing dissatisfaction/frustration with modern soccer in this regard. Similarly, the issue of a diminished stadium experience is cited (19), which participants in this study also describe under the category of poor quality of the stadium experience [see, for example (3, 7, 21, 22), regarding the aspect of soccer's diminished attractiveness]. Interestingly, participants in the sample also repeatedly mention the negative influence of other fans on their stadium visits in this context, as discussed by Hie et al. (25) [see also Kaden et al. (3) on the mutual influence between soccer fans]. The issues that participants cite in connection with traveling to and from the stadium are repeatedly mentioned not in isolation, but in relation to the personal circumstances of individual people (22). In particular, parallels can be drawn here to Tamir's (23) life-cycle approach, whereby life as a sports fan undergoes immediate and inevitable changes whenever one's personal life (outside of one's fandom) also changes.

**Figure 3:**
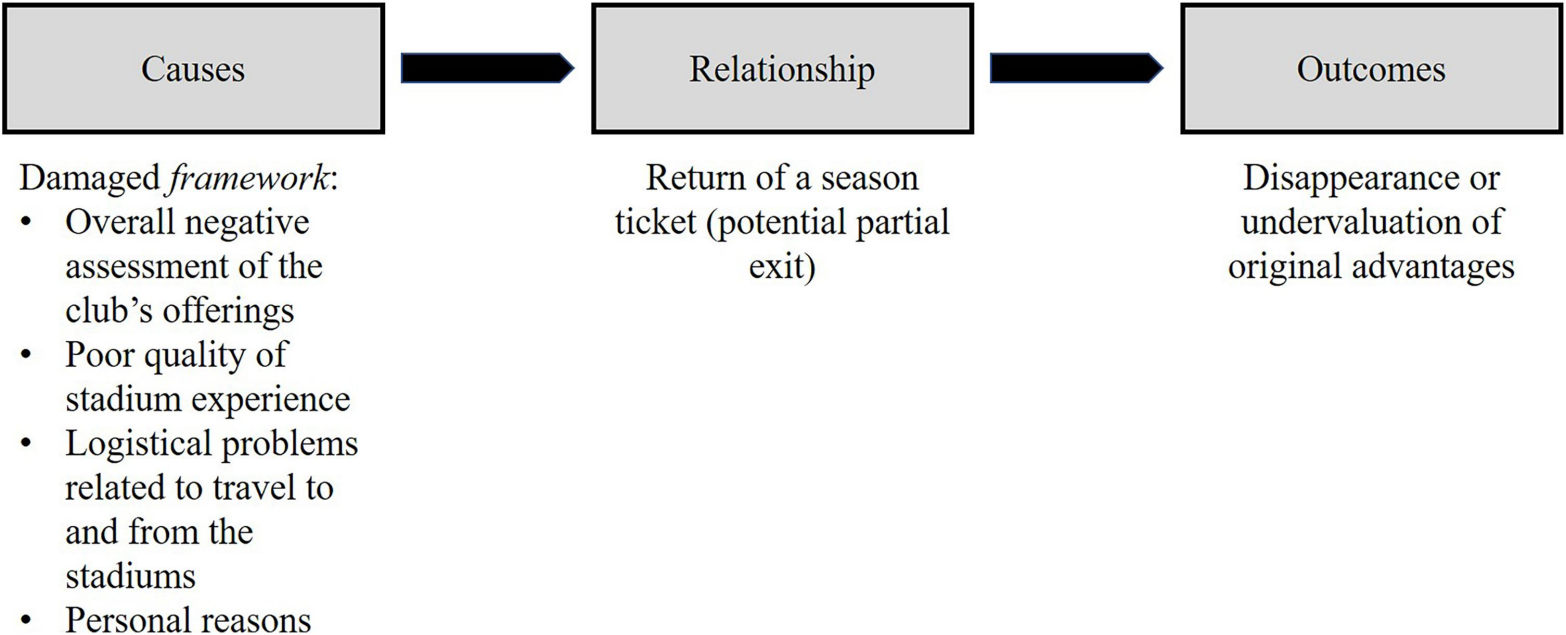
Fan–club relationship and return of season ticket.

The following overview places the results regarding returned season tickets in the context of the fan–club relationship (Figure 3). The diverse reasons for this behavior reflect less significant events and developments within the connection, but they certainly do reveal variations of glitches in the relationship framework between club and fans (without affecting the *foundation*), which can lead to partial withdrawal.

### 6.2 Theoretical implications

The results of the study suggest further implications for theory development of fan behavior and attitudes in professional sports, particularly regarding the theoretical constructs of fan exit and loyalty. The potential diversity of reasons that can be decisive for leaving or remaining in a relationship with a club calls for a more comprehensive conceptualization of attitudinal and behavioral fan loyalty and exit [building on the distinction introduced by Kaden et al. (3) between partial and total exit] as multidimensional and context-dependent phenomena. These conceptualizations should consider the identification of fans with, and emotional attachment to clubs under the influence of fan milieus, fans' professional and familial demands, and other factors mentioned above. Based on a comprehensive analysis of cases of abandoned memberships and non-renewal of season tickets (taking into account their relevant accompanying circumstances), the study identifies relevant differences. While non-renewal of season tickets appears repeatedly related to personal reasons and individual conditions of the fan experience, quitting a membership seems more related to criticism toward the club and its governance. In this regard, becoming a non-member tends to be more an expression of the general change of attachment to the club than becoming a non-season ticket holder. However, this is still an exploratory finding. In this regard, validation studies could be conducted, providing sufficient empirical data, which would allow these cases to be classified into the categories of partial and total exit, and promoting the operationalization of these constructs (as well as their delimitation). The resulting thoroughly differentiated constructs could also serve to enrich dynamic (life-cycle as well as life-phase-oriented) models of the relationships between fans and clubs (23) and increase their explanatory power.

Building on this, a further impetus concerns the *development of fan segmentation* in professional sports. Traditional segmentation approaches [like (11); for an overview, see, e.g., (12), p. 164 ff.; (13), p. 1117 ff.] often emphasize intensity-related characteristics of fans' sports consumption (e.g., regularity of stadium visits or media use in the case of sporting events). For a further development of segmentation approaches, it is advisable to consider more *motivation-* or *benefit-dependent (situationally differentiated) segmentation criteria*. For example, fans could be differentiated according to whether their sports-related behavior (concerning the given study, the status of season tickets and/or club memberships can be used as a relevant point of reference in this regard) is primarily shaped by individual life circumstances, social embeddedness in fan communities, normative attitudes toward professional soccer, or different preferences regarding the form of sports consumption via media or in the stadium. Such segmentations could also be integrated into the dynamic models of fan–club relationships mentioned.

### 6.3 Managerial/practical implications

Professional soccer clubs need to understand why fans give up their season tickets and/or memberships, thereby reducing or potentially ending their connection to the club to a certain degree. In this regard, various reasons can lead to superficially identical decisions, and these reasons sometimes occur in combination. However, without a deeper understanding of the specific reasons involved, it is not possible to make a definitive interpretation regarding a supporter's exit. Such information can be crucial for the club in identifying a (potential) decline in supporter loyalty at an early stage and in properly assessing its relationships with fans. Furthermore, for existing club management, this means that while metrics such as season ticket renewal rates or membership trends and developments remain important monitoring tools, their value is limited as long as the underlying causes of cancellations—considering the range of motivations fans associate with their relationship to the club—are not systematically tracked and analyzed.

Therefore, it is recommended that clubs systematically record reasons for cancellation, ideally at the moment of contract termination. An appropriate method would be to use standardized online *exit surveys*, which are conducted as part of the cancellation process. Based on the explorative results of this study, a standardized section should cover typical reasons for cancellation, such as changes in personal circumstances (e.g., relocation, work commitments), financial aspects (e.g., ticket prices, membership fees), sporting factors (e.g., valuation of team performance), criteria of club management and governance (e.g., dissatisfaction with management decisions), and elements of the stadium experience (e.g., atmosphere, security, or infrastructure). In addition, open-ended fields allow fans to explain their choice in more detail and address aspects not yet considered, as this study shows the relevance of qualitative insights (whereas standardized surveys often fail to capture all motivations in sufficient depth).

The collected information should be *compiled in a central database* (e.g., within a fan relationship management system). This allows cancellation decisions to be linked to other characteristics of the affected fans, such as membership duration, type of membership canceled (e.g., full membership, supporting membership), or season ticket, as well as the affected section of the stands, place of residence, or age of the fans. This, possibly taking into account existing (or newly determined) classifications of a club's supporters into defined subgroups, segments, or clusters [see also (11–13) in this regard], allows for the identification of patterns that may indicate different types of cancellations. When interpreting and drawing conclusions, it is crucial to distinguish between reasons for cancellation that clubs can influence and those over which they have no control. The latter includes factors such as health issues or changes in the personal situation of fans. Reasons that can be influenced, at least partially (e.g., ticket prices, evaluation of the stadium experience, transparency of club management, or fan involvement in decision-making processes), are particularly relevant for strategic member and fan management, as they provide concrete levers for performance-related, communicative, or organizational changes.

To support clubs in addressing the exit phenomenon in a goal-oriented and prioritized manner, it is recommended to categorize fan segments within a framework defined by (a) the *significance of the reasons* relevant to a fan group from the club's perspective (while maintaining the distinction between those that can and cannot be influenced) and (b) the *importance of a fan group* to achieving a club's goals (according to their financial and non-financial contributions). The analysis of these reasons focuses on those associated with a changed positioning from the fan's point of view, which hinders their identification with the club or team. If these fan segments are highly valued by the club (due to high revenues, margins, non-financial contributions, etc.), they should be given high priority in the development of measures. This underscores that the systematic analysis of cancellations should not be considered in isolation, but rather embedded in a holistic fan relationship management concept that allows for appropriate action/response by the clubs. The aim of such an approach is not only to understand past cancellation decisions, but also to identify potential risks for future cancellations at an early stage. Indications of declining loyalty can include, for example, season ticket holders rarely attending matches (33), members hardly taking advantage of club offerings, or repeated negative evaluations of certain club areas in surveys. If such signals are recognized early, clubs can take targeted measures to stabilize fan loyalty. Given the importance of this task, it is essential that clear responsibilities and accountabilities are defined for carrying out the aforementioned subtasks (e.g., by establishing dedicated departments).

### 6.4 Limitations and future research areas

The limitations of the study stem primarily from the data collection methodology. Since a number of professional clubs, contacted as the first step of the research approach, indicated that they could not comply with this request for data protection reasons, or showed little interest in participating in the study, participants were recruited through the social media platform Reddit, involving various subreddits. Since the questionnaire participants are likely to consist predominantly of active fans in this regard, this may introduce a certain degree of self-selection bias. Those enacting a (total) exit from fandom are probably not present in these Reddit threads [see also the limitations noted in the studies by Jha et al. (35) and Merten et al. (36), in which Reddit was also used for data collection]. It should also be noted that, although it can be assumed that the participants are active fans (including a corresponding degree of loyalty to their favorite clubs), the qualitative design of this study did not allow for a precise measurement or scale-based quantification of different levels of attachment. For further research on this topic, it is therefore necessary to conduct quantitative measurements of loyalty levels to empirically refine and extend the implications for action regarding the (potential) cancellation of season tickets and memberships.

In line with the suggestions made, the results also highlight the need for research to more precisely distinguish between *short-term changes* in fan behavior and *long-term processes* of fan disengagement (or even stabilization of fan–club relationships), which cannot be clearly differentiated on the surface. For example, if logistical problems lead to foregoing a season ticket, these may be short-term adjustments (which may need to be corrected quickly) that do not necessarily express a strong distancing from the club (and therefore require less fundamental responses from the club). Further research on fan behavior in professional sports must therefore systematically map such temporal differences in perspective and develop explanatory models that consider both endangered/impaired or stable relationships and temporary changes in fan engagement. Accordingly, longitudinal studies are warranted, as they incorporate a temporal dimension into the examination of the exit issue [see, for example, Kaden et al. (3) on the processual characteristics of increasing dissatisfaction among soccer supporters, or Bodon (24), and his phased extension of the Psychological Continuum Model].

## Data Availability

The raw data supporting the conclusions of this article will be made available by the authors, without undue reservation.

## Footnotes

^1^The rule ensures that even if a professional soccer team is spun off, the club and its members will still hold the majority of votes in the shareholders' meeting of the spun-off company (precisely 50 % plus 1 additional vote).
